# An Earnest Protest against the Control of Public Health Affairs of This Country by the Marine Hospital Service

**Published:** 1898-02

**Authors:** Warren P. Anderson


### An Earnest Protest Against the Control of Public Health Affairs of this Country by the Marine Hospital Service.

---

*Hon. Frank S. Gardner, Secretary New York Board of Trade and Transportation, New York City:*

Dear Sir.—In response to your inquiry concerning the efficiency of the Marine Hospital Service in the conduct of Federal health affairs, I forwarded you some days ago copies of the Journal of the American Medical Association, the *Florida Times-Union and Citizen*, and *New Orleans Times-Democrat*, as I was not able to reply immediately on account of certain professional engagements. I desire now to answer your queries briefly, but the presentation of such facts only as may not be successfully controverted, and which are detailed without malice towards any one or animus of a personal character whatever.

The first objection to be urged against such control of health affairs by this service or branch of the Federal Government is one relating to *organic* construction. You are doubtless familiar with the fact that only applicants between the ages of twenty-one and twenty-eight years are examined for admission to this service, and you may easily infer that ninety-eight per cent. of them have never had experience with epidemic diseases such as are likely to prevail at any time in our country. That they may be skilled in bacteriological lore I will readily admit; that they are without practical knowledge of any epidemic disease, they are, I am sure, also honest enough to confess. Thus, a young graduate from a Maryland, Michigan or Massachusetts medical college, standing an excellent but purely theoretical examination before the Board of Examining Surgeons, is dispatched by his Chief to preside over the health destiny of New

Orleans, Galveston or Mobile, with no more knowledge of the prevailing diseases or the commercial necessities of the people, than an Ashantee chief possesses of the military strength of Gibralta.

The second objection is largely an emphasis of the first, relating more nearly to the fact that these officials are taken indiscriminately from every part of the United States, less than five per cent. of them belonging south of latitude thirty-seven, or that area of the country likely to be invaded by yellow fever—our most common and most dreaded foe. Less than ten per cent. of the regular force of the service have had any experience with this disease, and less than three per cent. are regarded by sanitarians or the general public as possessing any knowledge of this disease whatever. Possibly two or three per cent. may have acquired immunity from the disease incident to the South, either by residence or a previous attack of such diseases. What a commentary upon the much advertised and magnificent equipment of this service for the health preservation of our country!

The third objection is one relating largely to the autocratic management of this service. Its officials are responsible to no one save the head of the Bureau at Washington, a Cæsarian system of government. See the utter disregard manifested for the health of the people of Georgia in 1893, when the medical officer of the service at the quarantine station sickened with yellow fever (the first victim), was removed to the principal hotel of Brunswick, where he died, and an epidemic of the dread disease followed. Witness the humiliating spectacle of Mississippi's delegation to the Federal Congress pleading in vain for the removal of a near and constant menace to the health of that beloved State—the Ship Island Quarantine Station. Then ask yourself if the Czar of all the Russias could lend so deaf an ear to the plaintive wails of his humble subjects.

The fourth objection is one based entirely upon the past inefficiency of this service as compared with the alleged weak control by the State. Twice in four years, at quarantine stations operated only by the authority of this service, yellow fever has gained entrance to our country, put to flight its people and ruined its commerce; whereas, under the management of the much despised State Boards of Health of quarantine stations, exclusively in their possession, no such invasion has occurred for nearly twenty years, save in Florida, and there not since the creation of the State Board of Health some nine years ago.

These are cold, stubborn facts, the proof of which, if desired' can be obtained from contemporaneous authorities or the archives of the Marine Hospital Service at Washington.

That this service is equipped in any manner, save in the possession of a magnificent bacteriological laboratory, for the protection of the people of the United States against the invasion of epidemic diseases, it were sheer folly to contend. That it is thoroughly equipped in this one particular, I am willing to believe, but I still maintain that the use of a few grains of common sense in the management of Federal quarantine matters is worth barrels of gelatine culture, uniquely prepared and labelled, for the inspection of the distinguished microscopist, while helpless communities are being ravaged by pestilence and panic.

It is not my object or purpose to hold the head of this Bureau, Dr. Wyman, responsible for its many defects, as I consider most of them organic in their character and far beyond the reach of any reform measure he might desire to institute. It is as impossible to unite a sailors' hospital service and the health affairs of this great country as to mix oil and water. The attempts of the future in this direction will show as dismal and disastrous failures as those of the recent past, and I believe the history of the world does not present a parallel to so gruesome and absurd a system. Let us try, however, to obtain a system wherein are combined the wisdom and judgment of the State in the matter of the appointment of its own citizens to these responsible positions, and the enormous power of the general government to control and direct the operations against the common enemy. Let us have a Marine Hospital Service and its capable ministrations to sick seamen, but let us also have a separate department of PUBLIC HEALTH, composed of men only of known ability and experience in such matters, whose sole object and aim shall be the promotion of the health interests of the entire country.

If this cannot be done, for one I shall prefer a continuance of the control over maritime quarantine by the State, to the exercise of these powers by a service so incongruous and so unsuccessful as this autocratic Bureau of the Treasury Department has demonstrated itself to be.

The idea of a Department of Public Health has taken a firm hold upon our people; from North, South, East and West come endorsements of such a general system, marked by many differences of opinion, it is true, as to minor details, etc. The press of the country, almost witout exception, commend such a course; commercial organization approve of it enthusiastically, and the two great bodies of advanced scientific thought on this continent, the American Medical Association and the American Public Health Association, have given it an unqualified endorsement. So far as my information extends, the sentiment in favor of the supervision or control of the public Health, both maratime and domestic, by the Federal government, is as overwhelmingly in favor of such a system as it is diametrically opposed to the management of the Marine Hospital Service, and I believe such opinions will assume a concrete form not differing materially from the ideas embodied in Senate Bill No 2343.

I desire to publish this communication, as I believe it more fully emphasizes my position than any other communication which I have written.

Very truly yours,

WARREN P. ANDERSON.

---

**At Least One Reason** for the "free and unlimited coinage" of testimonials for proprietary articles by St. Louis physicians has been disclosed lately. It is to secure patronage for the advertising pages of medical journals. A curious example is that of the *Medical Fortnightly*. This excellent journal (which has a sworn circulation of more than 7,000 copies each issue, but which until lately has been shipt from St. Joseph to St. Louis in ONE SMALL TRUNK every other week!) is presumably issued from St. Louis, tho' printed and now mailed at St. Joseph, and edited by Dr. Frank P. Norbury, of Jacksonville, Ill., the versatile and ultra-ethnical medical superintendent of Oak Lawn Retreat. It will be remembered that this is the gentleman who raised such a howl a few months ago because some stories, which he conceived to be vulgar, were told at the "smoker" of the Tri-State Medical Society; a man of his high character could not silently endure the thought that men like Lydston, Mink, Hughes and other famous reconteurs should tell risque stories at midnight's hour—it was too horrible to contemplate; and so he made a great protest that was echoed from Jacksonville to St. Joseph. But in securing advertisements for the *Fortnightly*, this highly moral editor is not so particular in regard to the element of truth; a lie is not half so bed as a smutty story. Strange philosophy; stranger religion! Strain at a tale that smacks of the pothouse; swallow a lie that would put Ananias to shame!

The latest attempt to secure advertising patronage read thus:

"Gentlemen:—In view of the fact that the writer has great personal confidence in your preparation, using it both in private practice and Oak Lawn Retreat, it would affords us great pleasure to bring your specialty to the notice of the physicians of the Mississippi valley.

"Please observe the sample copy sent you by this mail, noting the character of the institutions and the list of advertising patrons.

"We inclose herewith our net rates, and will accept one-third of the yearly contract in goods for use in Oak Lawn Retreat.

"We feel assured that representation in the *Fortnightly*, when reinforced by the writer's personal endorsement of your —— —— —— cannot fail to bring you good returns.

"Kindly let us hear from you, and if you contract for space for '98, we will accord you free insertion of the advertisement in the remaining issues of this year.

"Enclosed find some literature bearing upon the subject of 'quality and quantity' in circulation which it will pay you to examine. Yours very truly,

THE FORTNIGHTLY PRESS CO.,

FRANK P. NORBURY,

(Copy) Managing Editor.

Esau's sale of his birthright for a mess of pottage is not a fit comparison to the medical man, who sells his good name to advance the interest of a job printer who employs him. And to what base plans have medical publishers descended!—From *Lanphear's Journal of Surgery and Gynecology.*

